# Economic evaluation of severe malaria in children under 14 years in Zambia

**DOI:** 10.1186/s12962-022-00340-9

**Published:** 2022-02-05

**Authors:** Michael Mtalimanja, Kassim Said Abasse, James Lamon Mtalimanja, Xu Zheng Yuan, Du Wenwen, Wei Xu

**Affiliations:** 1 School of International Pharmaceutical Business, China Pharmaceutical University, Nanjing, 211198 Jiangsu China; 2 Faculté des Sciences de l’administration (FSA), Université Laval, Québec, QC G1V 0A6 Canada; 3 Department of Monitoring and Evaluation, Ministry of Health, P.O Box, 30205 Lusaka, Zambia

**Keywords:** Cost-effectiveness, Severe malaria, Quinine, Artesunate, Household income, Zambia

## Abstract

**Introduction:**

Malaria exerts a significant economic burden on health care providers and households and our study attempts to make claims on the cost effectiveness of artesunate against quinine in patients under 14 years of age in Zambia. Also, to find the average total costs involved in the treatment of severe malaria in children and their impact on household expenditure.

**Methods:**

Cost-effectiveness analysis of severe malaria treatment was conducted from a healthcare provider perspective using a Markov model. Standard costing was performed for the identification, measurement and assessment phases with data from quantification reports for anti-malaria commodities as these documents provides drug procurement costs from suppliers and freight costs. Average and incremental cost-effectiveness ratio were estimated and uncertainties were assessed through probabilistic sensitivity analysis.

**Results:**

In Zambia severe malaria in children has been shown to account for over 45% of the total monthly curative healthcare costs incurred by households compared to the mean per capita monthly income. The cost of treating severe malaria depleted 7.67% of the monthly average household income. According, to the cost effectiveness analysis the of artesunate with quinine the ICER was $105 per death averted.

**Conclusion:**

The use of artesunate over quinine in the treatment of severe malaria in children under 14 years is a highly cost-effective strategy for the healthcare provider in Zambia.

## Introduction

Malaria remains a major public health problem in Zambia, despite significant progress made in fighting the disease in the last decade. Malaria prevalence varies across all provinces and districts with 18 million people at risk, including the most vulnerable groups, such as pregnant women and children. The country’s last two iterations of the national malaria strategic plan (NMSP) aimed to reduce transmission through multiple strategies, including the distribution of long-lasting insecticide-treated mosquito nets (LLINs), increased indoor residual spaying (IRS), mass drug administration, improved case management using rapid diagnostic tests (RDTs)/microscopic laboratory tests, and treatment with artemisinin-based combination therapy [1].

In the current NMSP (2017–2021), the government of Zambia through the ministry of health and the national malaria elimination program (NMEP) adopted an ambitious agenda to eliminate malaria through deployment of the above outlined interventions, inclusion of new tools and innovations and strengthening of routine surveillance at all levels. The efforts towards nationwide malaria elimination with regard to malaria case management, emphasizes the need to have diagnostic and curative services as close to homes as possible while utilizing community health workers as extensions for the health facility within the community. In the recent past the NMEP has provided an annual sustained supply of more than 15 million treatment courses of the recommended artemisinin combination therapies and over 20 million rapid diagnostic tests. This is in addition to ensuring availability of more than 5.5 million tablets for intermittent presumptive treatment for pregnant women.

With an estimated 20.3% parasite prevalence, the NMEP has adopted therapeutic approaches such as mass drug administration (MDA) to accelerate the decline of parasite prevalence. On the other hand, case management coverage has greatly improved through strengthening of general health services and the provision of adequate diagnostics and medicines according to national guidelines [2]. The national objective is to ensure that 100% of all suspected malaria cases in all districts receive parasitological (microscopy or RDT) analysis and all parasitological confirmed malaria cases receive prompt (within 24 h), effective antimalarial treatment. Moreover, attaining universal coverage by providing service for all, with early diagnosis and effective treatment is a key strategy in reducing morbidity and mortality. The total malaria commodity needed to meet client needs per year with a full pipeline of 6 months of stock is estimated at around $ 27,374,448 which possess financial challenges [3]. Despite a better understanding of pathophysiology and management of malaria, childhood mortality remains unacceptably high [4]. Thus, over the past decade, there has been some progress in defining best practices for antimalarial treatment. The artesunate versus quinine in severe malaria in African children trial (AQUAMAT), conducted in 9 African countries and involving 5425 children, showed that artesunate-treated children having a 22.5% (95% confidence interval, 8.1 to 36.9) lower relative risk of death than those receiving the time-honored quinine [5]. Therefore, in 2011 the world health organization (WHO) recommended its use in preference to quinine as first‐line treatment for people with severe malaria. Prior to this recommendation many countries, particularly in Africa, had begun to use artemether, an alternative artemisinin derivative. Nevertheless, artesunate is recommended for treating adults and children that have severe malaria as studies have shown that it results in fewer deaths compared to treatment with quinine [6]. Notwithstanding, severe and fatal *Plasmodium falciparum* malaria continues to affect young children in sub-Sahara Africa representing approximately 90% of total global the cases, and one of the main causes of hospital admission and inpatient mortality [7].

Malaria exerts a significant economic burden on health care providers and households. Particularly, the total annual costs for malaria interventions in Ghana, Tanzania and Kenya were estimated at US$ 37.8 million, US$ 131.9million and US$ 109 million respectively. In addition, out of pocket towards treatment ranged from US$5.98 to US$45.23 for families [8]. Also, the cost of inpatient care for a case of severe malaria has been estimated between US$ 12 and US$ 75 which further exerts a heavy financial burden on most countries with already limited resources [9–11]. Most recently, many governments of the sub-Saharan region adopted plans to aggressively eliminate malaria in the region and sustained efforts towards malaria elimination in most of the countries have been seen to produce desirable results and thus in the right direction to attaining the intended goal.

Therefore, in the context of increasing attention towards improved malaria control in settings with budget constraints, competing health problems and weak health systems, it is essential to provide policy makers with relevant economic evidence of the economic benefits of health care control and prevention strategies. Despite having literature on sources of cost effectiveness of artesunate over quine in sub Saharan Africa and Southeast Asia, there is no single document that has attempted to make claims that it is a cost-effective strategy in the settings of Zambia. Thus, there is need to harness the fragmented information into one document thereby providing an opportunity to gauge and use the evidence to support the continual implementation of treatment interventions. In addition, the data on the costs involved in treating the condition exist in its raw form without linking the disease burden to household expenditure. The researcher believes that severe malaria related treatment costs, cannot be understood fully unless there is an attempt to discuss the disease burden in light of out of pocket payments. Therefore, our study attempts to make claims on the cost effectiveness of artesunate against quinine in Zambia. Also, to find the average total costs involved in the treatment of severe malaria in children and their impact on household expenditure.

## Methods

This study was designed to compare the costs involved in artesunate and quinine treatment regimen for severe malaria in children under 14 years, owing to this age group being at the highest risk of malaria infections and as well as to ascertain the household cost incurred in treating each episode. Products compared were injectable formulations, quinin 300 mg ampoule and artesunate 60 mg ampoule and also the costs relating to severe malaria treatment. Reference materials such as government publications, journal’s electronic database and conference papers were consulted to extract and provide information related to costs, utilities, transition probabilities and efficacy in the treatment of severe malaria. Search strategies included the generic drug names, brand names, efficacy, hazard ratios etc. Data collection was from 1st January to 31st October, 2020 and analysis was conducted in November of the same year.

### Model overview

**Fig. 1:**
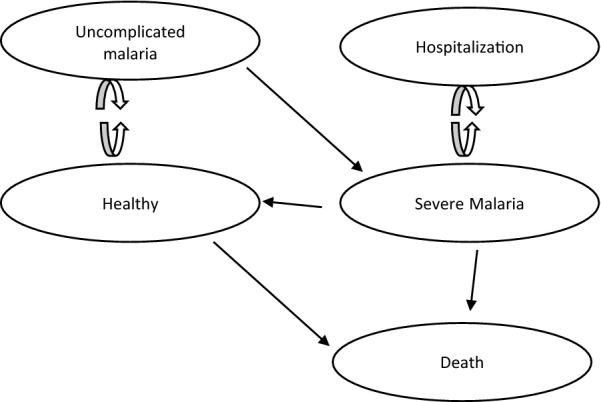
disease progression

We modelled the disease progression for severe malaria. Five main states of health were distinguished: (1) healthy or disease-free; (2) transition state of uncomplicated malaria; (3) severe malaria; (4) transition state of hospitalization; and (5) death from severe malaria. Model input data, included mortality rates, hazard ratio and possible transitions probabilities between health states. Thus, these were deterministic within the Markov cycles taking form of a disease progression where healthy children can either remain healthy, die or acquire uncomplicated malaria which progresses to severe malaria. Then, Children in severe malaria state get hospitalized and receive Artesunate or quinine. While children in severe malaria state either recover fully after treatment to enter the state healthy or die as shown in Fig. 1.

### Utilities

Due to scarcity of quality-of-life estimations in children affected by severe malaria, we used a self-administered visual analogue scale (VAS) based on the scale employed as part of the EQ-5D a standardized instrument that scores five health levels i.e., mobility, self-care, daily activities, pain/discomfort, and anxiety/depression whereas the patients/caregivers indicate experiences of their health status on a scale from 0 to 100. Also, to properly interpret the scale results, this was guided by trained medical staff, a methodology approach described by McCarthy et al. to estimate the age-specific quality-of-life utility weights for the different health states [12].

### Costs

The Zambia annual quantification report for anti-malaria commodities 2017–2018 was used to determine monthly costs of each treatment. This document was selected because it integrates both product cost and any applicable freight cost from all suppliers of the commodities, is readily available, and avoids the ambiguity of various product discounts and additional costs without freight charges from average landing cost. Other costs were obtained from hospital local procurement documents and local wholesalers and published literature [13]. Drug administration cost per dose of Artesunate and quinine included costs of a pair of examination gloves, 2 needles, 5 mg syringe and, 2 needles, 5 mg syringe, 1000 ml normal saline, IV infusion set respectively. Also, diagnostic costs were included in the model. Treatment costs for severe malaria were divided into pharmacological treatment, laboratory and nursing care. All cost conversions from Zambian kwacha to United States dollar was based on the exchange rate of October month end of the same year.

### Cost-effectiveness analysis

A cost-effectiveness model was constructed as a Markov model using stochastic parameters, created in Microsoft excel, with cycles having 1-year time and analysis taking the perspective of a healthcare provider. The model used beta distributions for treatment probabilities and utilities in view of the fact that it restricts values between 0.0 and 1.0 while gamma distributions were used for cost variables because of data skewness and also it is easily estimated from the mean and standard deviation i.e. the mean of the gamma distribution is αβ and the variance (square of the standard deviation) is αβ^2^ considering that alpha values = (mean)^2^/(standard deviation)^2^ and beta values beta = (standard deviation)^2^/(mean) respectively [14]. In addition, populations were created and followed until death to estimate costs and QALYs time horizon of 14 years in a Markov process with half cycle correction built into the analysis. Also, the model was employed to calculate the ICER for the case of a single cohort of 1000 children aged between 1 and 14 years who are healthy and prone to the high prevalence of malaria. As cost-effectiveness analysis is generally applied for a single cohort, these complementary results permit comparison with published data. The total costs of direct and non-direct medical costs of the two arms artesunate and quinine as well as the variables used in the model are as shown in Table 1.

**Table 1:** Costs and model values

	Model value	Mean	Stdev	Alpha	Beta	Distribution type	Formula	Source
Mortality								
Age < 14 years	0.11	0.11						[26]
Severe malaria	0.23	0.36						[31]
Transition probabilities								
Healthy-death	0.11							[26]
Disease-death	0.23							[31]
Healthy-disease	0.03							[13]
Hospitalization	0.98							[13]
Drug costs								
Artesunate drug	7.8							
Other drugs	3.6							
Fluids	10.7							
Laboratory tests	6.4							
Nursing care	37.1							
Total drug cost	65.6	66	9	30,625	0.0000229	Gamma		[24, 32–34]
Quinine drug	2.6							[24, 32, 35]
Other drugs	3.8							
Fluids	11.0							
Laboratory tests	6.6							
Nursing care	37.4							
Total drug cost	61.4	61	6	225,000	0.0001333	Gamma		
Cost hospitalization	65	65	19	11.7036011	6	Gamma		[24, 36, 37]
Hazard ratio								
HR die artesunate	0.78	64	0.01	–	–	Normal		[22, 38]
HR disease artesunate	0.64	64	0.01	-	–	Normal		[22, 38]
HR hospitalization artesunate	1.02	1	0.001	–	–	Normal		[22, 38, 39]
Utility								
Utility healthy	0.98	0.98	0.07	3	0	Beta		[12, 40, 41]
Utility disease	0.80	0.80	0.05	50	13	Beta		[12, 42, 43]
Utility decrement hospitalization	0.1	0.1	0.08	1	12	Beta		[13, 43]
Discount rate	5.0%	5.0%	–	–	–	–	–	[1]

### Probabilistic sensitivity analysis

In any economic evaluation such as this there are several key variables that are subject to uncertainty. A probabilistic sensitivity analysis was in excel using Monte Carlo simulation to assess the effect of uncertainty surrounding the costs and effectiveness estimates. Each variable was allocated a distribution fitting the range of all possible values with each simulation randomly generating and select the value for each variable from the specified distribution. Consequently, examining the effect of joint uncertainty in the variables of the model through cost-effectiveness plane and acceptability curve. The cost-effectiveness plane shows the incremental cost on the vertical axis and effectiveness on the horizontal axis for 1000 simulation runs. Also, results showed the mean value and 95% confidence intervals (CI) for total costs and QALYs. Sensitivity analysis allows exploration of the impact of change in one or more of these variables on the result robustness [15].

## Results

According to our research, the outcomes indicated on average households spend $ 23.45 for an episode of severe malaria accounting for over 45% of the total monthly curative healthcare costs incurred by households compared to the mean per capita monthly income. On the other hand, the cost of treating severe malaria depleted 7.67% of the monthly average household income. Costs involved in each severe malaria episode for children under 14 years revealed on average about $10.5 was incurred by the caregivers’ due to productivity loss days of work and $7.75 for direct medical costs. This represented approximately 26% of the mean per capita monthly income. While registration fee, consultation fee, drugs/surgical, diagnostic tests and transportation representing $0.75, $4.5, $2, $0.5, and $5.2 respectively as shown in Table 2.

**Table 2:** Household income expenditure

	Variables	Minimum average cost	Maximum average cost	Average cost (SD)	Percentage age mean-per capita monthly income %
Direct medical cost	Registration fee	0.15	4.25	0.75 (0.65)	1.9
	Consultation fee	4.24	17.5	4.5 (4.1)	11.3
	Drugs/surgical	0.25		2.0 (1.8)	5
	Diagnostic tests (RDT/Laboratory)	0.5	2.5	0.5 (0.3)	1.3
Sub-total				7.75	19.3
Direct non-medical cost	Transportation	2.0	6.5	5.2 (2.7)	13.0
Indirect cost	Loss of income			10.5 (0.28)	26.25
Overall cost				23.45	48.75

Table 3 shows the incremental cost-effectiveness ratios were estimated as the total healthcare cost per death averted. Our study, revealed that the strategy of using Artesunate over quinine has an ICER of $91 (95% CI 71.6–153.4) per death averted. This represents the average incremental cost associated with preventing one unit of death related to severe malaria in children under 14 years in Zambia.

**Table 3:** Incremental cost effectiveness ratio

Treatment	Cost	QALY	Incremental		ICER
Artesunate	409.41	5.99	69.09	0.76	90.82
Quinine	340.32	5.22			

**Fig. 2:**
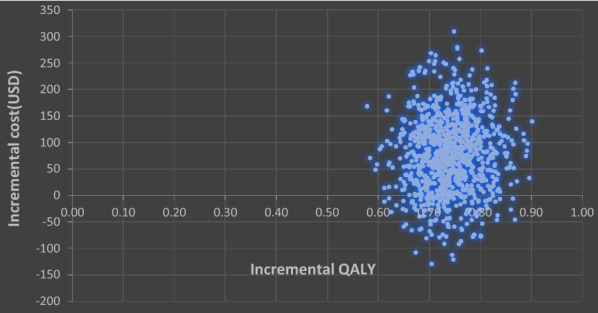
Incremental cost-effectiveness ratio (ICER) scatterplot

Scatter plot of 1000 samples of mean incremental costs plotted against mean incremental effectiveness was generated for the two drug therapies. The most of the point were found to lie in the upper right-hand quadrant, indicating increased costs and increased effectiveness with a few points in the lower right-hand quadrant indicating cases being dominant in the stimulation as shown in Fig. 2. Thus, the graph shows an additional investment in terms of cost would produce an extra unit of the desired health outcome hence the intervention being cost-effective.

**Fig. 3:**
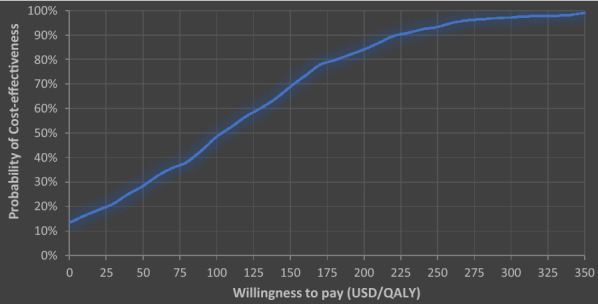
Cost-effectiveness acceptability curve

According to the base-case acceptability curve for artesunate therapy versus quinine therapy generated from 1000 samples of mean incremental costs versus mean incremental effectiveness. The acceptability curve showed how likely one therapy is cost-effective for any particular willingness to-pay with our study revealing the probability of artesunate being cost-effective being approximately 12% without any additional investment. In addition, with a willingness-to-pay of $150 and $300, artesunate produces a probability being cost-effectiveness of 70%, and above 95% respectively as in Fig. 3.

**Fig. 4:**
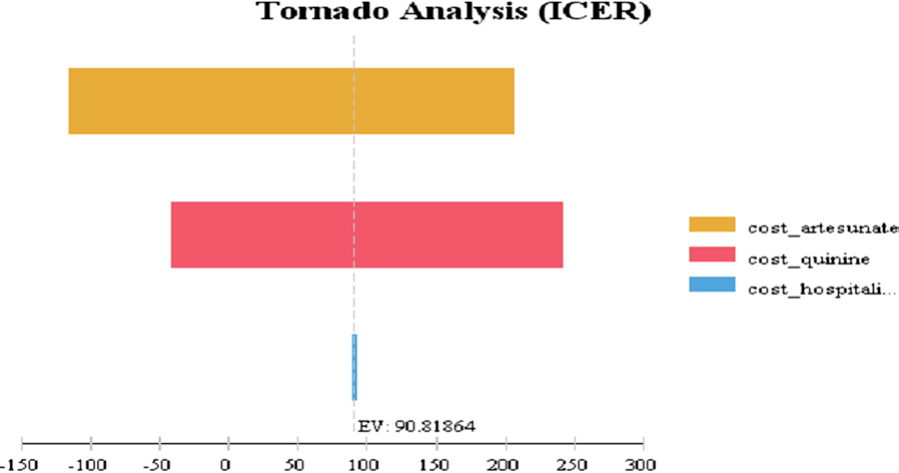
Sensitivity analysis

Conforming to Fig. 4, One-way sensitive analysis demonstrated that cost-effectiveness was most sensitive to drug costs of artesunate followed by costs of quinine and cost of hospitalization respectively. Varying the mean costs by ± 30% resulted in the ICER ranging from dominant (-$110) to ($240) per QALY of death averted.

## Discussion

Pertaining to treatment of severe malaria and full implementation of injectable artesunate use in public and private hospitals, governments require effective communications on its costs and benefits in the context of each country for a clear definition of the projected national funding requirements and availability of financial resources. Thus, after conducting our cost-effectiveness analysis, the model revealed artesunate arm costing $65.6 which compares favorably to the mean costs from studies by Lubell et al. who recorded $66.5 and 61.4 for the quinine arm respectively [16]. Similarly, the outcomes were lower than those earlier reported from a research conducted in Zambia which showed a cost of US$77 for severe malaria episodes and also slightly above the average range (1.4–65) in a separate study in Kenya [17, 18].

Our model-based analyses suggested health benefits associated with the use of artesunate in children with severe malaria being cost-effective when compared with the use of quinine at commonly accepted willingness-to-pay threshold. In addition, treatment indicated increased costs and increased effectiveness as well as some cases showing dominance, implying that in some cases the treatment costs less and with increased effectiveness than the comparator drug. Moreover, the authors lead to the belief of its use could significantly have cost savings through avoided drug administration costs and nursing care to alleviate risk of cardiotoxicity, as intravenous quinine administration needs rate-controlled infusion over 4 h, three times a day, accompanied by cardiac monitoring if possible [19]. Also, a study examining malaria related deaths showed one in four patients on quinine received incorrect dosing potentially resulting into increased drug adverse effects cost and death [20]. Consequently, due to high mortality rate among children, the benefits of expanded use of artesunate could be a right step in the right direction to reduce the malaria burden [21]. The cost of averting malaria-related deaths in Zambia by switching from quinine had an ICER value of US$ 91 per death averted**.** The results are similar to the incremental cost per death averted in children in sub-Saharan African, estimated on average to be US$123 [5, 22, 23]. This is seen to correspond well to other interventions, such as the use of insecticide-treated nets, with a cost per death averted of US$ 254 to US$ 3437 [24, 25]. Not only artesunate is cost effective but simpler to administer and reduces episodes of hypoglycemia during treatment by 45% hence a cost saving therapy and consequently its use in the management of severe malaria in children is seen to have more monetary benefits [21]. Furthermore, the robustness of our results over a range of varying assumptions was tested in the sensitivity analysis, even with conservative estimates around the parameters used in the model for sensitivity analysis, the findings remain cost-effective across a range of estimates in the model on assumptions at the threshold of willingness to pay similarly used in other studies of 3 times the gross domestic product per capita as an upper threshold and recognized by the world health organization [23, 26].

In Zambia like many other countries medical services are not covered 100% by the state and citizen suffer a great deal on out of pocket payments. In view of household expenses on malaria, expenses can be classified into expenditure on prevention and expenditure on treatment which include direct payment of drugs, consultation, laboratory tests, transportation fees to and from the health facility, and the caregiver’s productive time lost due to malaria. Thus, seeking treatment poses a serious impact on household medical expenditure. Our research outcomes indicate a slightly higher average cost of $23.45 for each episode of severe malaria compared to the report by the central statistical office of Zambia in the 2015 living condition monitory survey reporting on average one spending $12 towards medication or consultation, while rural/urban analysis indicated on average $8 and $19.5 respectively [27]. Transportation costs accounted for an average of $5.2 and 13% of the mean per capita household income even though healthcare access is still associated with higher care seeking costs due to long distances to health facility and the mode of transportation used. Overall costs for management of severe malaria episodes are similar to those observed in Malawi and Mozambique [28, 29].

Households bear a greater portion of this cost due to high level of indirect costs resulting in spending more than the estimated monthly total expenditure, to some households this may be catastrophic as a result of low mean per capita monthly household income in Zambia of $40 which is defined as the total household income divided by the number of persons in the household [30]. Hence, there is need to buffer this with some sort of financial risk protection mechanisms and the health care system strengthened to function more effectively and decrease overall out of pocket payments to aid in alleviating economic burden of malaria on the general population. Hence the need for government to sustain the provision of free malaria treatment [27].

Our assessment had considerable limitations that are expected in the construction of any decision model. Firstly, societal perspective of economic evaluation has a more comprehensive framework for analysis but we took a healthcare perspective because malaria treatment is free of charge from all government hospitals. Also, our assumptions were that of a patient having only one episode of severe malaria during the one-year cycle.

Owing to the fact that cost effectiveness models can be sensitive to time horizon of the analysis, and in most cases covering a life expectancy time horizon. In the case the incremental cost comparisons may be somewhat accurate, but the cumulated incremental benefits may be significantly underestimated.

In addition, the retrospective design to collect data on household costs potentially would cause recall bias, owing to patients not accurately remembering costs involved in the malaria treatment. Thus, the costs in our study may underestimate the true household costs for severe malaria episodes.

## Conclusion

The artesunate therapy is highly cost-effective treatment for severe malaria in Zambia and anticipated to significantly reduce the current mortality caused by the disease with a simpler route of administration. Also, findings of this study contribute to the evidence focusing attention on the substantial economic burden of severe malaria episodes on households.

## Data Availability

The data set used/analyzed are available from the corresponding author on request.

## Abbreviations

DHIS: District Health Information System
ICEAR: Incremental cost effectiveness ratio
IRS: Indoor Residual Spraying
LLINs: Long Lasting Insecticide Nets
MACEPA: Malaria Control and Elimination Partnership in Africa
MOH: Ministry of Health
NMEP: National Malaria Elimination Program
PMI: President’s Malaria Initiative
RDTs: Rapid diagnostic tests
USAID: United States Agency for International Development
WHO: World Health Organization
